# Performance of High-Dose Reclaimed Asphalt Mixtures (RAPs) in Hot In-Place Recycling Based on Balanced Design

**DOI:** 10.3390/ma17092096

**Published:** 2024-04-29

**Authors:** Lei Jiang, Junan Shen, Wei Wang

**Affiliations:** 1 Jiangsu Technology Industrialization and Research Center of Ecological Road Engineering, Suzhou University of Science and Technology, Suzhou 215011, China; jl951329414@163.com; 2 Department of Civil Engineering and Construction, Georgia Southern University, Statesboro, GA 30458, USA; 3 School of Civil Engineering, Chongqing Jiaotong University, Chongqing 400074, China; wwangcqjtu@outlook.com

**Keywords:** high RAP content, laboratory-accelerated aging test, hot in-place recycling asphalt mixtures, road performance

## Abstract

This study endeavors to employ a balanced design methodology, aiming to equilibrate the resistance to rutting and cracking exhibited by hot in-place recycling asphalt mixtures containing a high dose of reclaimed asphalt pavement (RAP). The primary goal is to ascertain the optimal amount of new binder necessary for practical engineering applications, ensuring a balanced rutting and crack resistance performance of recycled asphalt mixtures. The investigation mainly employed wheel-tracking tests and semi-circular bending tests to assess the rutting and cracking performance of recycled asphalt mixtures with a different dose of RAP (in China, it is common to use RAP with 80% and 90% content as additives for preparing hot in-place recycling asphalt mixtures), and varying quantities of new binders (10%, 20%, and 30% of the binder content in the total RAP added). The results indicated that the addition of new binder reduced the resistance to rutting of the recycling asphalt mixtures but improved their resistance to cracking. Furthermore, for the recycling asphalt mixture with 80% RAP content aged for 5 days, the optimal new binder content is 1.52%, while the mixture with 90% RAP content requires 1.23% of new binder. After 10 days of aging, the optimal new binder content for the recycling asphalt mixture with 80% RAP content is 1.55%, while the mixture with 90% RAP content requires 1.28% of new binder.

## 1. Introduction

Flexible pavements often experience various types of on-site damage due to factors such as prolonged and increased vehicle loading and severe climate fluctuations. Rutting and cracking are two of the common damages on flexible pavements as a result, which not only reduce the smoothness of the road surface but also affect driving comfort and safety. The performance of pavement materials is a critical factor contributing to these damages. To improve pavement material performance, adjustments to the composition or the development of new performance evaluation standards and design methods can be implemented.

Recycled/reclaimed asphalt mixture (RAP) has gradually gained widespread adoption in the highway industry, with an increased dose of RAP in recycling mixtures, enabling the 100% recycling of reclaimed asphalt pavement road surfaces, yielding multiple benefits. Firstly, it contributes to the conservation of natural aggregates and binder, among other natural resources. Secondly, it effectively reduces the emission of industrial waste, thereby mitigating environmental pollution. Additionally, recycling asphalt mixture helps lower energy consumption [1,2]. The recycled material, HMA, exhibited superior mechanical and resilient modulus performances. Furthermore, higher tensile strength ratios of the recycled material mixtures indicated a greater resistance to water damage [3]. However, due to durability issues associated with a high dose of RAP in recycling asphalt mixtures, and considering factors such as fatigue and water damage, many countries impose restrictions on the maximum allowable RAP content [4,5]. Currently, in hot in-place recycling technologies, the percentage of RAP in the asphalt mixture is relatively high but generally does not exceed 70–80%. During the mixture design phase, the primary consideration is that the aged binder should fully dissolve and mix with rejuvenating agents or new binder during the mechanical blending process, aiming for the complete regeneration of the aged binder. However, studies indicate that achieving this premise is challenging in practical engineering applications [6,7,8,9,10]. Therefore, the recycling degree of RAP plays a crucial role in the preparation of hot recycled mixtures, and current recycling methods lack such an essential characterization in this aspect [11]. Furthermore, the rejuvenating degree of RAP depends on its degree of aging, presenting another crucial factor, i.e., the fusion recycling level between the aged RAP binder and the newly introduced virgin binder. Recycling of aged RAP binder is considered an effective form of binder that forms a new binder mortar, bonding the aggregates together to create recycled asphalt mixtures [12]. Researchers categorize the mixing scenario between RAP and asphalt mixtures into three possible situations: (a) all RAP binder is mobilized and mixed with the original binder; (b) no RAP binder is activated, and RAP acts like black aggregates; and (c) some RAP binder is activated and mixed with the virgin binders [13,14,15].

Therefore, in designing recycling asphalt mixtures, it is necessary to better understand the mixing phenomenon and adjust the design procedures accordingly. Some studies have introduced an intrinsic property called the Degree of binder Activity (DoA) and redefined two well-known concepts, namely the Degree of Blending (DoB) and the Degree of binder Availability (DoAv). Practical recommendations have been proposed to incorporate them into the mixture design process [16].

Traditional design methods such as Superpave and Marshall methods typically determine the optimal binder content for mixture design based on volumetric parameters and estimate mixture performance. However, they do not guarantee the performance of asphalt mixtures. These methods have high requirements for material performance, especially for the stability, deformation resistance, and durability of binders. However, the situation of binder in high-dose RAP is complex, and the intrinsic properties of aged binder (DoA) as well as the DoB and DoAv of aged binder with rejuvenating agents are not yet fully known at this point. The cracking of asphalt pavement during use is often one of the primary factors affecting the service life of asphalt pavement designed by conventional methods [17].

Nowadays, there is an increasing emphasis on utilizing performance-based evaluation for asphalt mixture design and tests. The main aim of these tests is to employ established performance specifications to identify substandard asphalt mixtures, such as those prone to rutting or cracking, at various stages of the asphalt mixture design or production. Several studies have proved the ability of such tests to assess the performance of asphalt mixtures [18,19,20,21,22,23,24,25,26,27,28,29,30,31,32,33,34,35].

In 2006, Zhou et al. [36] introduced the Balanced Mix Design (BMD), a method aimed at finding a balance between rutting and cracking resistance to determine the optimum binder content of asphalt mixtures. Feng et al. [37], in 2011, applied rutting tests and low-temperature flexural beam tests for the balanced design of asphalt mixtures regarding cracking and rutting resistance. In 2012, Walubita and Hu et al. [38] conducted comparative experiments using the Accelerated Loading Facility (ALF) system, comparing asphalt mixtures designed by the Texas Department of Transportation (TxDOT) method with those designed by the Balanced Mix Design. The results demonstrated that asphalt mixtures designed by the Balanced Mix Design method exhibited superior performance. In the same year, Zhou et al. [39] first applied the Balanced Mix Design to RAP mixtures to determine an optimal binder content.

This study endeavors to investigate high-percentage hot in-place recycling asphalt mixtures, specifically focusing on augmenting the proportion of RAP content. Through the application of performance-balanced design, a specific quantity of supplementary new binder is incorporated to offset the unactivated fraction of aged binder present in RAP, thereby guaranteeing superior resistance to rutting and cracking in the recycling asphalt mixture.

To attain this goal, this study employed dense-graded asphalt mixture AC-13 and prepared different levels of aged RAP mixtures through laboratory short-term and long-term aging processes. The research entails the formulation of recycling asphalt mixtures incorporating RAP content levels of 80% and 90% with different dosages of virgin binders. Subsequently, this study assesses the rutting and cracking resistance performance subsequent to the incorporation of diverse quantities of new binder.

## 2. Laboratory Experimental Program

**Figure 1:**
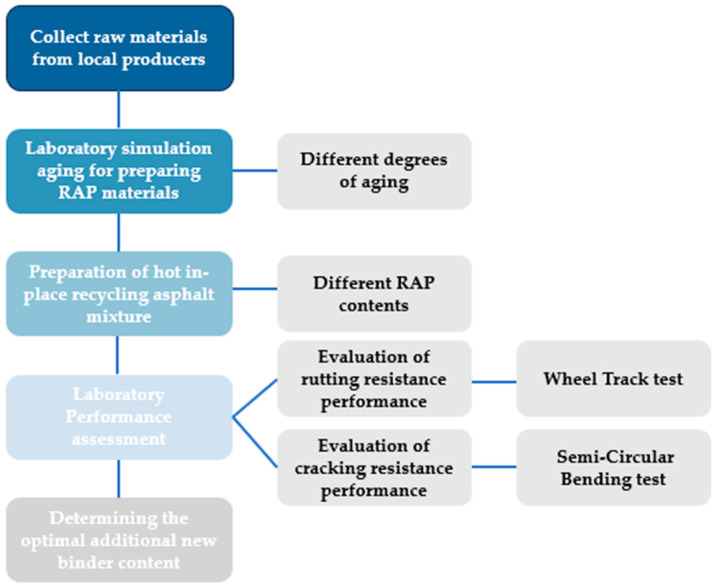
The experimental plan.

Figure 1 illustrates the experimental plan of this study. Firstly, raw materials (i.e., binder, aggregates, and rejuvenating agents) were collected from China, Suzhou, Suzhou Sanchuang Road Engineering Co., Ltd. Then, laboratory-aged asphalt mixtures were prepared using the accelerated aging process at different aging levels (5 days and 10 days). Next, the preparation of hot in-place recycling asphalt mixtures with different lab-prepared RAP contents (80% and 90%) was carried out. Subsequently, representative tests for evaluating the cracking and rutting performance were selected. Finally, based on the concept of BMD, the optimal additional binder content was determined. The following sections discuss the cracking and rutting performance tests selection, properties of raw materials, sample preparation, and testing procedure.

### 2.1. Properties of Raw Materials

#### 2.1.1. Binder

Utilizing styrene–butadiene–styrene (SBS)-modified binder, the key technical specifications are presented in Table 1. These properties are determined by the supplier. Testing methods were all conducted in accordance with the specifications [40].

#### 2.1.2. Mineral Material

The basalt aggregate used in this study is detailed in Table 2, which provides the particle size distribution for each aggregate gradation and mineral powder. The properties for both coarse and fine aggregates, as well as mineral powder, meet the specifications as required.

#### 2.1.3. Rejuvenator

The primary component of the rejuvenating agent is low-viscosity mineral oil derived from petroleum. Its main function is to replenish the saturated and aromatic fractions lost during the aging process of binder, aiming to restore some of the pavement performance of modified binder and secondarily recycled mixtures. Therefore, essential characteristics of a high-quality rejuvenating agent include appropriate viscosity, favorable rheological properties, sufficient aromatic content, and a lower ratio of thin film oven test viscosity.

The technical specifications of the rejuvenating agent used in this study are presented in Table 3. These properties are determined by the supplier. It must be noted that the dose of rejuvenator is very efficient to soften the aged binders. The dose should be determined according to the target (i.e., PG grade or penetration) of the recycled binders. Based on our previous laboratory research results, the dose of this project incorporated into the recycling asphalt mixture is 5% of the binder content in the RAP [41].

### 2.2. Lab Preparation of RAP

**Figure 2:**
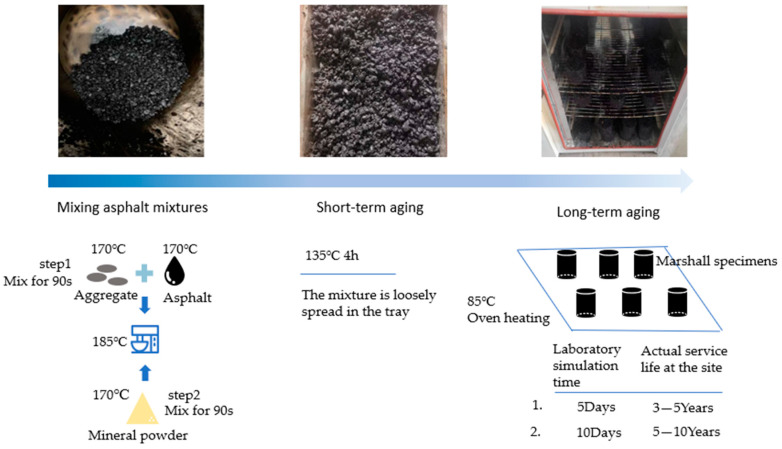
The aging steps (delete one of the layers to save space).

Accelerated aging was conducted according to JTG E20-2011 [40]. The aging steps are as shown in Figure 2. The aggregates were placed into the mixing pot heated to 185 °C, after being heated to 170 °C in an oven. After mixing aggregates with SBS-modified binder for 90 s, the mixing machine was paused to add the preheated mineral powder. Mixing continued for another 90 s to obtain the SBS-modified asphalt mixtures. The specimens must undergo short-term aging before long-term aging. The mixtures were first spread and placed in an oven at a temperature of 135 °C for 4 h under forced ventilation conditions as short-term aging. Then, the asphalt mixture was compacted into Marshall specimens and placed in an oven at a temperature of 85 °C. It underwent continuous heating for 5 days (assumed 3–5 years’ aging in the field) and 10 days (assumed 5–10 years’ aging) under forced ventilation conditions as long-term aging [40].

### 2.3. Lab-Prepared Hot In-Place Recycling Asphalt Mixture

**Figure 3:**
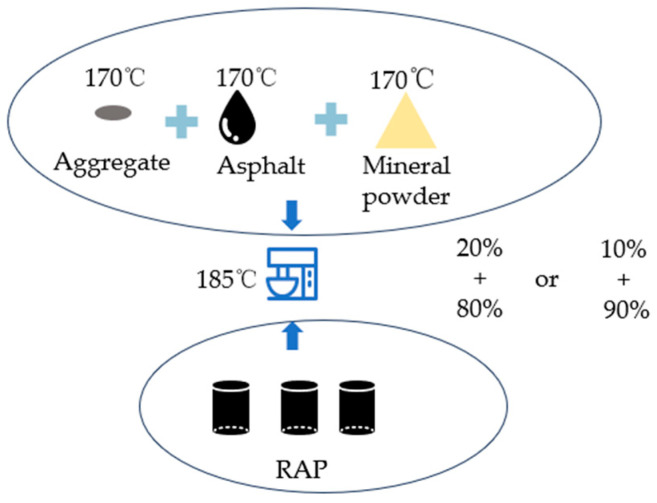
The process of preparing hot in-place recycling asphalt mixture.

The preparation process is illustrated in Figure 3. In the hot in-place recycling asphalt mixture, due to the presence of RAP, the RAP should be preheated at 170 °C for 2 h before mixing the recycling asphalt mixture. For optimal results, the rejuvenating agent should be mixed first with the old material during the mixing process. This helps soften and disperse the old material while promoting uniform mixing between the new and old materials. According to research findings, extending the mixing time can improve the performance of recycling asphalt mixtures in actual engineering applications. The laboratory mixing pot is simple in structure, but based on previous research experience, it is necessary to mix RAP with the rejuvenating agent for 120 s to achieve uniform mixing of the hot recycling asphalt mixture. Then, the preheated new aggregates are added to the mixing pot and mixed for 90 s. Subsequently, the preheated new binder, heated to 170 °C, is added and mixed for another 90 s. Finally, the preheated mineral powder is added and mixed for 90 s.

This study selected two different RAP blending percentages (80% and 90%) with the aim of increasing the utilization rate of RAP in actual engineering applications. The issue arising from high percentages of RAP utilization is the impact on the availability of aged binder during the recycling process. This study addresses this issue by incorporating additional amounts of new binder (i.e., 10%, 20%, and 30% of the binder content in the RAP).

The samples are grouped and labeled according to different variables, as shown in the Table 4:

Group A represents the recycling asphalt mixture (RAP) prepared with 80% RAP content after five days of aging. Groups B, C, D, and so on follow in sequence.

#### 2.3.1. Mix Proportion

**Figure 4:**
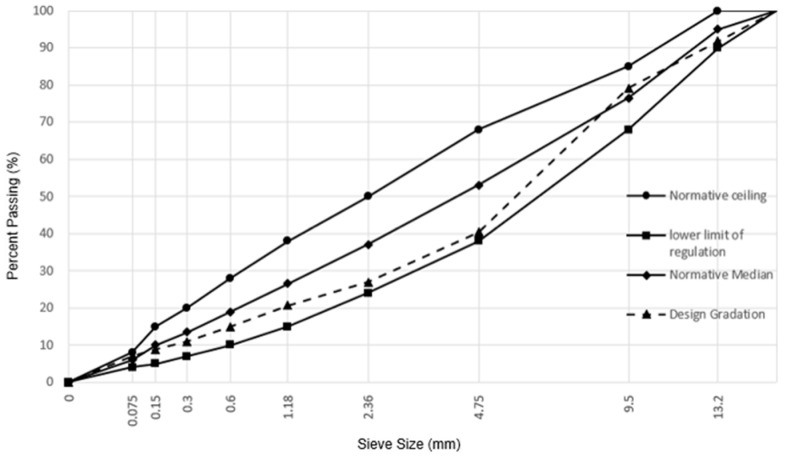
The gradation curves of recycling asphalt mixtures.

As the RAP used in this study underwent simulated aging in the laboratory, the damage and loss of aggregates during its aging process are controllable. Under both RAP content levels, the gradation curves remain consistent. The gradation curves of the recycling asphalt mixture are shown in Figure 4.

#### 2.3.2. The Binder Content of Recycling Asphalt Mixture

The binder content was experimentally obtained, and the binder content of the AC-13 conventional hot mix asphalt mixture was around 4.7%. This study took 4.7% as the initial estimated binder content value and calculated the initial estimated new binder content value (Pnb) based on the RAP content. Formed specimens were prepared at three different levels of new binder content: 10%, 20%, and 30% of the aged binder content in the RAP (control group at 0% RAP). The optimal new binder content value was determined using the balanced design method. The aged binder content extracted from the RAP was determined to be 4.6%. According to Equation (1), the estimated new binder content values for 80% and 90% RAP contents were calculated, as shown in Table 5.

(1) $$P_{nb}=P_{b}-P_{ob}\times \frac{n}{100}$$

where P_nb_ is the estimated new binder content (%), P_b_ is the total binder content of the hot recycling mixture (%), P_ob_ is the aged binder content in the RAP material (%), n is the RAP material mixing rate (%).

### 2.4. Selected Performance Tests and Indicators

#### 2.4.1. Performance Assessment Tests

In this study, the authors selected the two most representative tests for cracking and rutting resistance: the semi-circular bending (SCB) test and wheel track test.

**Figure 5:**
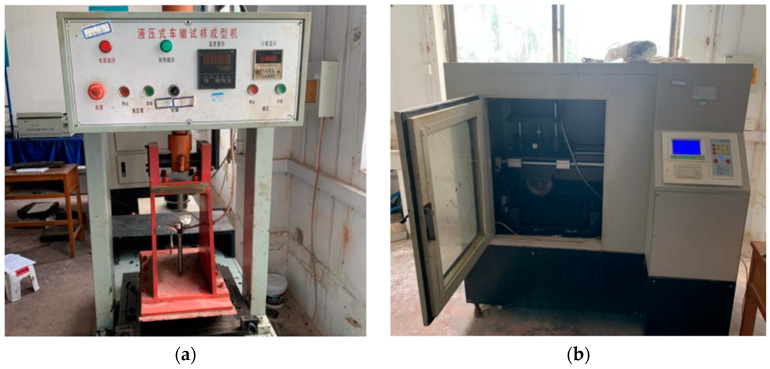
The rutting test equipment: (**a**) rutting sample forming machine, (**b**) wheel-track test machine.

The wheel-track test, conducted according to JTG E20-2011, forms rutting test specimens with dimensions of 300 mm × 300 mm × 50 mm using a rolling method. After forming, the specimens were placed at 60 °C for at least 5 h but not exceeding 12 h. The specimens, along with the test mold, were placed into the wheel-track testing machine. The contact pressure between the test wheel and the specimen was set at 0.7 ± 0.05 Mpa, the test temperature was maintained at 60 °C, and the rolling duration was 60 min with a rolling speed (N) of 42 times per minute. The shaping apparatus and the rutting tester are depicted in Figure 5. Each sample group was tested with four identical samples, totaling 64 samples.

The semi-circular bending test was conducted according to the reference [42]. The recycling mixtures were molded into Marshall specimens. Circular specimens with a thickness of 50 mm were sawed from the middle of the Marshall specimens, and then symmetrically split open from the middle to obtain semi-circular specimens. Finally, a notch was cut at the bottom of the semi-circular specimens with a depth of 10 mm and a width of 2 mm, thus obtaining the semi-circular bending specimens [43,44,45,46]. Each sample group was tested with four identical samples, totaling 64 samples.

**Figure 6:**
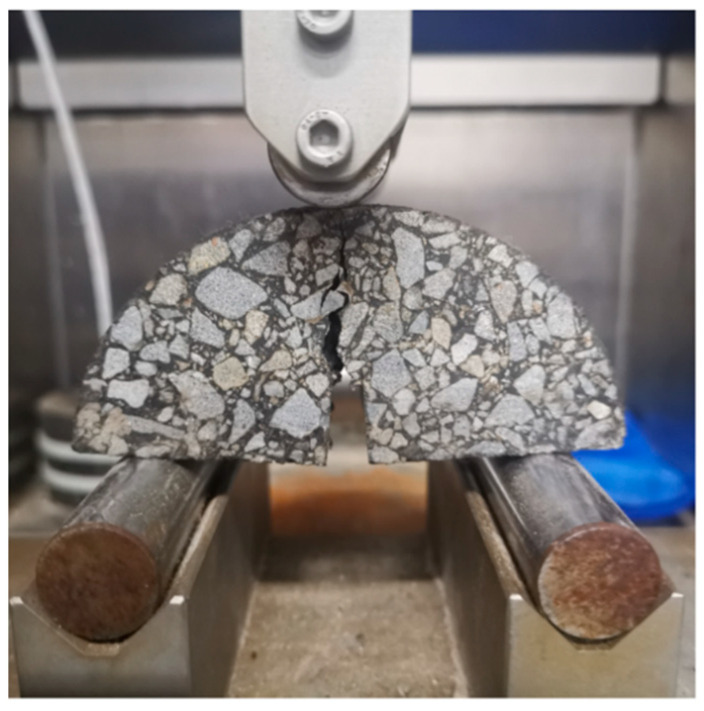
Semi-circular specimen test set.

Before the test, the specimens were placed in a constant temperature chamber and kept at 15 °C for 4 h. Subsequently, the specimens were symmetrically placed on supports with a spacing of 8 cm, and the test was conducted under the set temperature and loading rate conditions. The test was terminated when the specimen failed. The semi-circular bending strength test was conducted in the UTM130 multifunctional material testing system. To avoid impact failure, it was necessary to ensure contact between the upper loading head and the specimen before loading. Once the contact force stabilized at around 0.1 KN, loading of the specimen began until failure. The displacement change data at the mid-span loading head were automatically recorded using a linear variable displacement transducer (LVDT), while the change data of the vertical pressure and vertical displacement were recorded using an automatic data acquisition instrument. Figure 6 shows the semi-circular specimen during the test.

#### 2.4.2. Performance Assessment Indicators

The performance evaluation index for rutting resistance is the dynamic stability, calculated according to the Formula (2) specified in JTG E20-2011 T0719-2011. It reflects the deformation degree of the asphalt mixture under the action of external forces during high-speed driving. On highways, vehicles exert significant dynamic loads on the road surface. If the dynamic stability of the asphalt mixture is insufficient, deformation, looseness, or damage may occur, thereby affecting the service life of the pavement and driving safety. The formula is as follows:

(2) $$DS=\frac{\left(t_{2-}t_{1}\right)\times N}{d_{2}-d_{1}}\times C_{1}\times C_{2}$$

where DS is the dynamic stability (times/mm), t_1_ is 45 min, t_2_ is 60 min, d_1_, d_2_ is the deformation amount (mm) corresponding to time t_1_, t_2_, C_1_ is the coefficient for the type of testing machine, which operates in a reciprocating manner driven by a crank-link mechanism and is 1.0, C_2_ is the coefficient for the specimen, which is prepared in the laboratory with a width of 300 mm and is 1.0, N is the reciprocating rolling speed of the test wheel, which is usually 42 times per minute.

According to the “Technical Specification for Highway Asphalt Pavement Construction: JTGF40-2004” [47], the dynamic stability of the modified asphalt mixture should be greater than 3000 times/mm.

The performance evaluation index for crack resistance is the flexibility index (FI). “FI” reflects the flexibility and crack resistance of the asphalt mixture. In cold climates, the road surface is susceptible to temperature fluctuations. If the asphalt mixture’s crack resistance is insufficient, cracks and damage are more likely to occur, thereby affecting the pavement’s service life and driving safety. The calculation Formulas (3)–(6) are as follows:

(3) $$G_{f}=\frac{W_{f}}{A_{lig}}$$

where G_f_ represents the fracture energy, J/m^2^; W_f_ represents the work of fracture, J, A_lig_ represents the ligament area, m^2^.

The calculation formulas of W_f_ and A_lig_ are as follows: (4), (5)

(4) $$W_{f}=\int_{a}^{b}Pdu$$

where P represents the load, N; u represents the deformation, m

(5) $$A_{lig}=(r-a)\times t$$

where r represents the SCB specimen radius, m; a represents the depth of the pre-cut, m; t represents the thickness of SCB specimen

(6) $$FI=\frac{G_{f}}{\left|m\right|}A$$

where |m| represents the absolute value of the post-peak slope, m; A represents the unit scaling factor, which is recommended to be 0.01 in the IL-SCB test specification.

According to the research by Imad L and Al-Qadi et al. [27], it was found that when FI is greater than 4, the cracking rate of the mixture significantly decreases. Additionally, based on field trial results, it was observed that the FI values for AC-type mixtures with poor crack resistance are concentrated in the range of 1.3 to 3.9.

#### 2.4.3. Designing Balanced Asphalt Mixture (BMD)

Balanced Asphalt Mixture (BMD) is a method for designing asphalt mixtures, which involves conducting performance tests on specimens subjected to various distress conditions under appropriate conditions, taking into account traffic, climate, mixture aging, and specimen position in the pavement structure. In essence, BMD comprises two or more mechanical tests, such as rutting and cracking tests, to assess the performance of mixtures against common pavement distresses [48].

The information obtained from material characterization and performance testing is then utilized in the optimization phase of BMD. This phase identifies the range of binder content that yields the desired performance characteristics [49]. FHWA has developed and improved four types of balanced design methods: (A) volumetric design with performance verification, (B) volumetric design with performance optimization, (C) performance-modified volumetric mix design, and (D) performance design.

This study adopted the “D” method. Initially, the synthetic gradation of the aggregate was determined, and an estimated value for the new binder content was obtained. Performance tests were conducted using the new binder addition content. The results of the rutting and cracking tests were plotted on the same graph, identifying the new binder content point that simultaneously satisfies both the rutting and cracking requirements.

## 3. Results and Discussions

### 3.1. Evaluation of Rutting Resistance Performance

**Figure 7:**
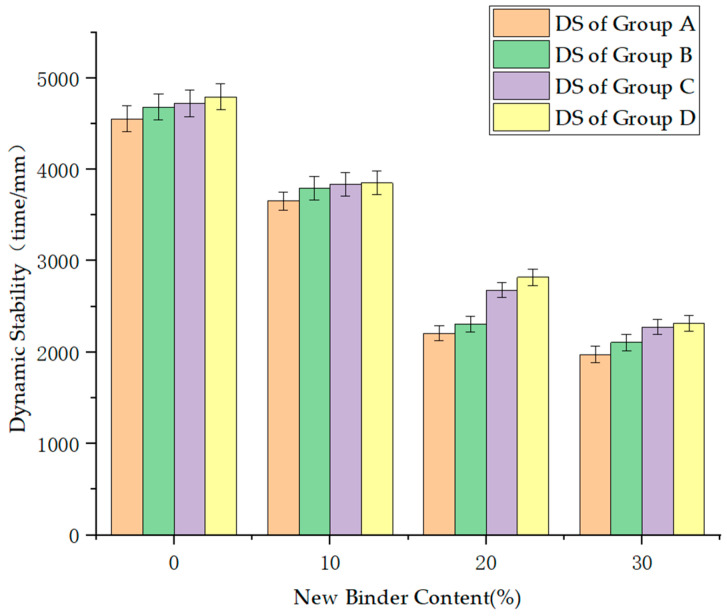
Dynamic stability of the hot in-place recycling asphalt mixtures.

The dynamic stability of the hot in-place recycling asphalt mixtures is illustrated in Figure 7.

According to the results shown in Figure 2, as the additional new binder content increases from 0% to 30%, the dynamic stability decreases. This variation can be explained by the increasing viscosity of the binder with the addition of new binder, leading to a weakening of the adhesion performance between the binder and aggregates. As a result, the mixture becomes more prone to shear under stress, reducing the dynamic stability. Simultaneously, the filling effect between the binder and aggregates intensifies, but it also reduces the contact area between the aggregates, increasing the gaps between the binder and aggregates, resulting in a looser mixture structure that is unfavorable for maintaining the dynamic stability [50,51,52].

Comparing the data between Group A and Group C, it can be observed that the dynamic stability has improved by 2.85% to 6.63%. Similarly, comparing the data between Group B and Group D, an improvement in the dynamic stability by 0.36% to 5.34% is shown. This indicates that with the deepening of aging, it is advantageous for the rutting resistance performance. The reason for this is that with the longer aging time, the binder undergoes oxidation and deterioration, altering its properties and enhancing the deformation resistance of the mixture [53].

Comparing the data between groups A and B, it can be observed that the dynamic stability improved by 3.67% to 21.40%. Similarly, comparing the data between groups C and D, the dynamic stability showed an improvement of 1.50% to 22.23%. This indicates that with the increase in the RAP content, the resistance to rutting of the recycling mixture improves [54,55]. The reason for this is that the higher content of aged binder in the RAP leads to greater viscosity, resulting in reduced plastic deformation at high temperatures.

In this experimental study, the dynamic stability of hot in-place recycling asphalt mixtures is influenced by factors such as the amount of new binder, the degree of RAP aging, and the content of RAP. These factors do not affect the dynamic stability of hot in-place recycling asphalt mixtures in exactly the same way, and further research is needed to investigate whether there are interactions among these factors. This paper employs multiple regression analysis to examine the impact of various factors on the dynamic stability of hot in-place recycling asphalt mixtures, and to predict their patterns of change. The aim is to understand the interactions among multiple factors and to provide feasible methods for improving the resistance to rutting of hot in-place recycling asphalt mixtures.

This paper conducts a multivariate regression analysis of the dynamic stability of hot in-place recycling asphalt mixtures under the influence of multiple factors using IBM SPSS Statistics 27.0.1 software. Prior to the regression analysis, it is necessary to address the issue of multicollinearity among the various factors in the data analysis, in order to avoid situations where multiple independent variables exhibit high correlation or linear correlation.

From Table 6, it is evident that the VIF values for each factor are all 1, much less than 5, indicating the absence of multicollinearity among variables. This suggests that a regression model for the dynamic stability of hot in-place recycling asphalt mixtures can be established and utilized for multiple linear regression calculations.

Table 6 reflects the linear correlation between various factors and the dependent variable. The correlation coefficient R^2^ represents the degree of fit of the model. Three factors—the amount of new binder, the degree of RAP aging, and the content of RAP—account for 95.4% of the variation in the dynamic stability of hot in-place recycling asphalt mixtures.

In terms of significance P, the significance of the amount of new binder P is less than 0.05, while the significance P of the aging degree and RAP content is greater than 0.05. Moreover, the significance value of the RAP aging degree, 0.460, is greater than that of the RAP content, 0.071, indicating that the effect of the RAP aging degree on the dynamic stability is minimal (insignificant and can be ignored), while the effect of the additional new binder content is maximal. Based on the above analysis, the regression Equation (7) can be derived as follows:

Y = 2329.425 − 88.395 × X1 + 2515.000 × X2(7)

In the equation, Y represents the dynamic stability, time/mm; X1 denotes the amount of new binder, %; and X2 signifies the content of RAP, %.

**Figure 8:**
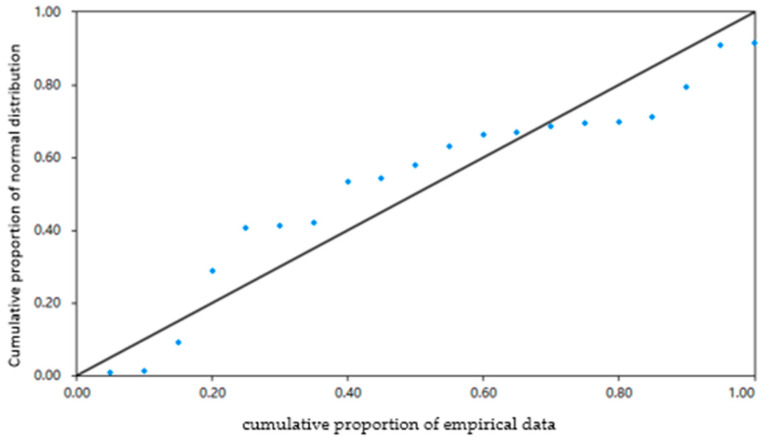
Percent–Percent Plot of dynamic stability.

The Percent–Percent Plot (P-P Plot) is commonly used to visually inspect whether data follow a normal distribution. Its principle lies in the fact that if the data are normally distributed, the cumulative proportion of the data should closely match the cumulative proportion of a normal distribution. This is achieved by plotting the actual cumulative proportion of the data on the *X*-axis against the corresponding cumulative proportion of a normal distribution on the *Y*-axis, creating a scatter plot. If the scatter plot approximately forms a diagonal line, it indicates that the data follow a normal distribution. Conversely, if it deviates from a straight line, it suggests that the data are non-normal. From Figure 8, it can be observed that the scatter points are mostly along the diagonal line, suggesting that the residuals of this regression model follow a normal distribution.

### 3.2. Evaluation of Cracking Resistance Performamce

**Figure 9:**
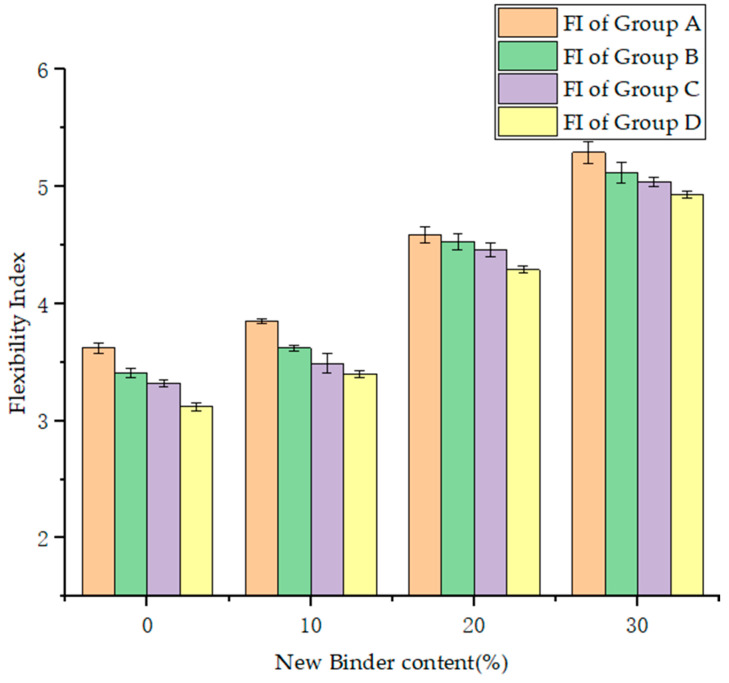
The flexibility index of the hot in-place recycling asphalt mixtures.

The flexibility index of the hot in-place recycling asphalt mixtures with the addition of different amounts of new binder are illustrated in Figure 9.

Based on Figure 9, it can be observed that with the increase in the binder content, the recycling asphalt mixture exhibits several positive effects, leading to a gradual increase in the flexibility index. The increased binder content may result in the formation of more binder films on the surface of the aggregates, improving the bonding performance between the binder and aggregates and helping to slow down the cracking of the mixture. Lastly, the higher binder content increases the flowability and deformability of the binder, leading to an increase in the elastic modulus of the mixture [27,56,57]. This makes the mixture more adaptable to external stresses, aiding in better shape retention and slowing down crack development. The combined effect of these factors results in positive improvements in low-temperature performance and resistance to cracking for the recycling asphalt mixture.

The phenomenon of embrittlement occurring after binder aging, accompanied by a decrease in the elastic modulus, makes the mixture more prone to cracking, leading to a continuous deterioration in the cracking resistance as reflected by the flexibility index. Comparing the data of Group A with Group C, it can be observed that the flexibility index decreases by 1.31% to 5.97%. Similarly, comparing the data of Group B with Group D, the flexibility index shows a decrease of 2.18% to 6.02%.

The addition of reclaimed materials can worsen the cracking resistance of the mixture. In particular, for recycling mixtures with a high proportion of reclaimed materials, their cracking resistance is significantly affected by the dosage. Comparing the data of Group A with Group B, it can be observed that the flexibility index decreases by 2.83% to 9.35%. Similarly, comparing the data of Group C with Group D, the flexibility index decreases by 3.71% to 8.50%.

According to the analysis method described in Section 3.1, similarly, how the flexibility index of the asphalt mixture is affected by the amount of new binder, the degree of RAP aging, and the content of RAP will be analyzed. The results are shown in Table 7.

From Table 7, it is evident that the VIF values for each factor are all 1, much less than 5, indicating the absence of multicollinearity among variables. This suggests that a regression model for the flexibility index of hot in-place recycling asphalt mixtures can be established and utilized for multiple linear regression calculations.

Table 7 reflects the linear correlation between various factors and the dependent variable. The correlation coefficient R^2^ represents the degree of fit of the model. Three factors—the amount of new binder, the degree of RAP aging, and the content of RAP—account for 95.6% of the variation in the flexibility index of hot in-place recycling asphalt mixtures.

From the perspective of significance levels P, the significance levels of the amount of new binder and RAP content are both less than 0.05. Additionally, the significance level of the RAP content (0.013) is greater than the significance level of the amount of new binder (0.001). However, the significance level of the RAP aging degree (0.094) is greater than 0.05, indicating that the effect of the RAP aging degree on the flexibility index is minimal, while the effect of the additional binder content is the most significant. Based on the above analysis, the regression Equation (8) is derived as follows:

Y = 5.557 + 0.061 × X1 − 0.031 × X2 − 2.475 × X3(8)

In the equation, Y represents the flexibility index; X1 denotes the amount of new binder, %; X2 signifies the degree of RAP aging, days; and X3 signifies the content of RAP, %.

**Figure 10:**
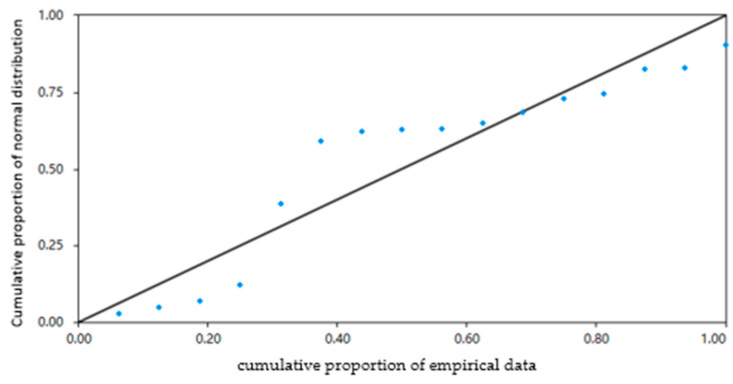
Percent–Percent Plot of flexibility index.

From Figure 10, it can be observed that the scatter points are mostly along the diagonal line, suggesting that the residuals of this regression model follow a normal distribution.

### 3.3. Determining the Optimal Binder Dosage

**Figure 11:**
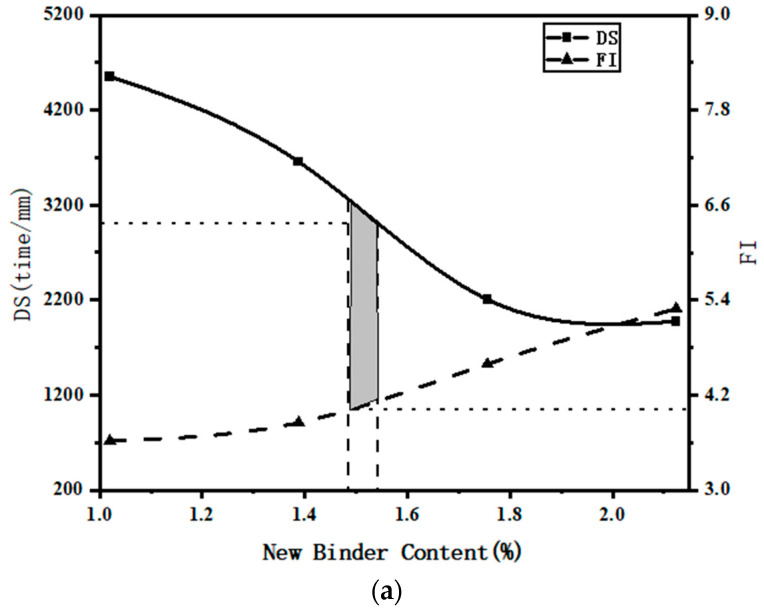

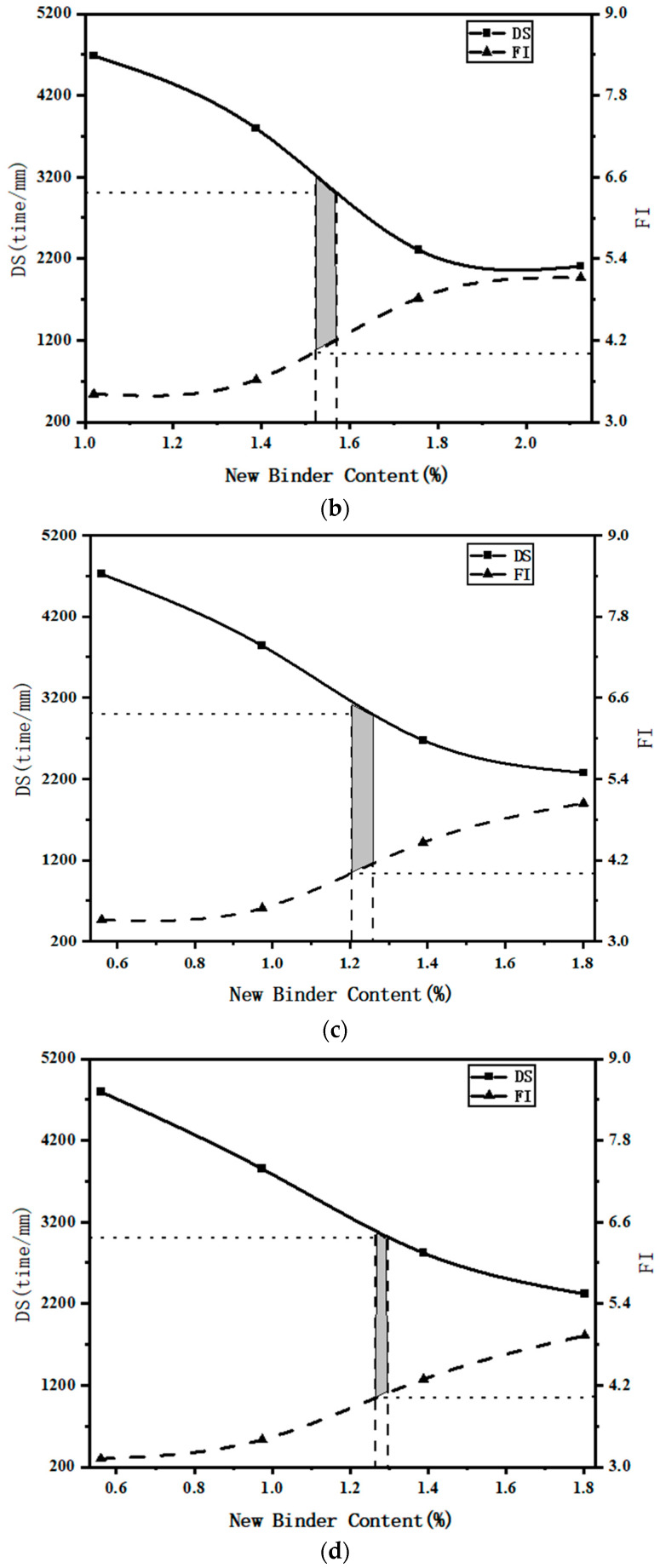
Binder content range chart for asphalt mixtures: (**a**) binder content of Group A, (**b**) binder content of Group B, (**c**) binder content of Group C, (**d**) binder content of Group D.

To visually present the experimental results, the test results of different hot in-place recycling asphalt mixtures are plotted as dual-axis line graphs, as shown in Figure 11. The grey area in the graph represents the range of new binder content values that meet the performance test criteria (dynamic stability > 3000 times/mm and FI > 4, according to Section 2.4.2).

The equilibrium-designed binder content graph for Group A is depicted in Figure 11a. The new binder content corresponding to a dynamic stability of 3000 times/mm is 1.54%. The new binder content corresponding to an FI value of 4 is 1.49%. Therefore, the optimal new binder content for the asphalt mixture obtained using the equilibrium design method is 1.52%. The summary of the experimental results for the different test groups is shown in Table 8.

From Table 8, it can be seen that the performance of the aged binder in the RAP is poor. As the RAP content increases, the balance design method requires more new binder to improve the cracking resistance performance of the recycling mixture, which leads to a continuous increase in the total binder content of the recycling asphalt mixture. As the aging time increases, the degree of binder aging deepens, leading to an increase in the total binder content and the addition of new binder in the recycling asphalt mixture. Prolonged aging makes the mixture more prone to embrittlement, reducing its viscosity and elasticity, thereby decreasing its resistance to cracking. To achieve a balance between resistance to rutting and resistance to cracking, it is necessary to increase the new binder content appropriately.

## 4. Conclusions and Recommendations

The main objective of this study is to utilize the concept of balanced design by balancing the rutting and cracking resistance of a recycling asphalt mixture, in order to determine the optimal new binder content for a high-dose RAP hot in-place recycling asphalt mixture in engineering applications. The conclusions are as follows:

(1) With the increase in the additional new binder content, the cracking resistance of the recycling asphalt mixture has improved. Significant differences exist between the recycling asphalt mixture with a high new binder content (such as 20% and 30%) and the control group (0%).
(2) As the additional binder content increases, the rutting resistance of recycling asphalt mixtures significantly decreases. With each 10% increase in new binder content, the dynamic stability of recycling asphalt mixtures decreases by nearly 19%. Additionally, the addition of new binder can enhance the crack resistance of high-dose RAP mixtures, but it reduces their rutting resistance, especially at high levels of new binder addition. For instance, when an extra 20% of new binder is added, the crack resistance of recycling asphalt mixtures increases by 27%, but their rutting resistance decreases by 51%.
(3) The dynamic stability and flexibility index of the mixture exhibit a strong linear relationship with the dosage of new binder, the aging degree of RAP, and the dosage of RAP (R2 = 0.954, 0.956). Among these factors, the dosage of new binder has a significant impact.
(4) Based on the balanced design of rutting and cracking resistance, the optimal new binder content for the Group A recycling asphalt mixture is 1.52%; for Group B, it is 1.55%; for Group C, it is 1.23%; and for Group D, it is 1.28%. As the RAP content increases, the rejuvenated aged binder positively influences the performance, reducing the amount of new binder added. However, the unactivated aged binder also increases, requiring more binder to meet the demand for crack resistance, leading to an overall increase in binder usage. With deeper binder aging and decreased viscosity, additional new binder is needed to restore the crack resistance, further contributing to the overall increase in binder consumption.
(5) Adding additional new binder to a high dose of hot in-place recycling asphalt mixtures effectively enhances their crack resistance. However, since the RAP used in this study was prepared in the laboratory, future work should consider linking laboratory design with field experiments to balance the design of actual construction site RAP. This will advance the performance balance design of in-place recycling asphalt mixtures with a high RAP content in the highway industry.

## Figures and Tables

**Table 1:** Main properties of binder.

Item	25 °C Penetration Degree/(0.1 mm)	Softening Point/°C	5 °C Ductility/cm	Residue after RTFOT		
				Mass Change/%	Penetration Ratio/%	5 °C Residual Ductility/cm
Specification	30–70	>65	>20	≤±1.0	≥65	≥15
Test results	54	78	36	0.22	73	25
Test methods	T0604	T0606	T0605	T0609	T0604	T0605

**Table 2:** The particle size distribution of the aggregates used in the mix design.

Sieve Size/mm	Percentage Passing/%.				
	10–15 mm	5–10 mm	3–5 mm	0–3 mm	Mineral Powder
16	100.0	100.0	100.0	100.0	100.0
13.2	62.8	100.0	100.0	100.0	100.0
9.5	11.5	96.6	100.0	100.0	100.0
4.75	0.2	7.1	93.6	100.0	100.0
2.36	0.2	0.2	5.5	83.5	100.0
1.18	0.2	0.2	1.6	62.1	100.0
0.6	0.2	0.2	1.1	41.7	100.0
0.3	0.2	0.2	1.1	27.8	100.0
0.15	0.2	0.2	1.1	20.0	100.0
0.075	0.2	0.2	1.1	13.8	98.3

**Table 3:** The technical specifications of the rejuvenating agent.

Test Item	Test Results	Technical Requirement
60 °C viscosity/(mm^2^·s^−1^)	59.2	50~175
Flash point/°C	242	≥220
Saturated fraction/%	17.31	≤30
25 °C density/(g·cm^−3^)	1.017	Actual measurement
Film oven viscosity ratio	1.22	≤3
Quality change in film oven/%	−1.174	≤4, ≥−4
Appearance	Brown-black viscous liquid	

**Table 4:** Grouping of recycling asphalt mixtures.

	RAP Content	80%	90%
Degree of Aging			
5 Days		A	C
10 Days		B	D

**Table 5:** Estimated new binder content values.

Estimated Total Binder Content/%	RAP Content/%	Aged Binder Content/%	Estimated New Binder Content/%	New Binder Addition Content/%
4.7	80	4.6	1.02	1.02
				1.388
				1.756
				2.124
4.7	90	4.6	0.56	0.56
				0.974
				1.388
				1.802

**Table 6:** Results of the linear regression analysis on dynamic stability.

Model	B	*p*-Value (P)	Variance Inflation Factor (VIF)	R^2^
Constant	2329.425	/	/	0.954
The amount of new binder	−88.395	0.001	1.000	
The degree of RAP aging	19.350	0.460	1.000	
The content of RAP	2515.000	0.071	1.000	

**Table 7:** Results of the linear regression analysis on flexibility index.

Model	B	*p*-Value (P)	Variance Inflation Factor (VIF)	R^2^
Constant	5.557	/	/	0.956
The amount of new binder	0.061	0.001	1.000	
The degree of RAP aging	−0.031	0.094	1.000	
The content of RAP	−2.475	0.013	1.000	

**Table 8:** Summary of experimental results.

RAP Content/%	Degree of Aging/Days	New Binder Content When FI = 4/%	New Binder Content When DS = 3000 times/mm/%	Optimal New Binder Content/%	Total Binder Content/%
80	5	1.49	1.54	1.52	5.20
80	10	1.52	1.57	1.55	5.23
90	5	1.20	1.26	1.23	5.37
90	10	1.26	1.30	1.28	5.42

## Data Availability

Data are contained within the article.
